# Stroke prevention in atrial fibrillation: A narrative review of current evidence and emerging strategies

**DOI:** 10.1111/eci.70082

**Published:** 2025-06-07

**Authors:** Amir Askarinejad, Deirdre A. Lane, Parham Sadeghipour, Majid Haghjoo, Gregory Y. H. Lip

**Affiliations:** ^1^ Liverpool Centre for Cardiovascular Sciences at University of Liverpool, Liverpool John Moores University and Liverpool Heart & Chest Hospital Liverpool UK; ^2^ Department of Cardiovascular and Metabolic Medicine Institute of Life Course and Metabolic Medicine, University of Liverpool Liverpool UK; ^3^ Department of Clinical Medicine Aalborg University Aalborg Denmark; ^4^ Rajaie Cardiovascular, Medical, and Research Center Iran University of Medical Sciences Tehran Iran; ^5^ Medical University of Bialystok Bialystok Poland

**Keywords:** 4S‐AF framework, ABC pathway, atrial fibrillation, digital health, left atrial appendage occlusion, oral anticoagulation, stroke prevention

## Abstract

**Background:**

Atrial fibrillation (AF), the most prevalent cardiac arrhythmia, is associated with a significantly increased risk of mortality and morbidity from stroke, thromboembolism and dementia. Recent advances in stroke prevention strategies necessitate an updated approach to management.

**Results:**

Published evidence shows that the Atrial Fibrillation Better Care (ABC) pathway significantly improves stroke prevention outcomes in AF patients, reducing mortality, stroke incidence and bleeding events. Characterisation of AF using the 4S‐AF framework helped guide personalised treatment selection and was associated with improved clinical outcomes. For patients unsuitable for anticoagulation, left atrial appendage occlusion has been identified as a viable alternative. Digital health technologies demonstrate increasing utility in early AF detection to enable timely stroke prevention interventions. There is evidence for the dynamic nature of stroke (and bleeding) risk, as well as arrhythmia burden and AF progression over time, in addition to changes in ABC pathway adherence.

**Conclusions:**

Effective stroke prevention in AF requires a comprehensive holistic approach incorporating appropriate risk stratification, guideline‐adherent anticoagulation and management of underlying cardiovascular conditions and other comorbidities. The ABC pathway, supported by characterisation using the 4S‐AF framework, provides a structured approach to optimise outcomes. Regular reassessment of risk, along with careful selection of anticoagulation strategies, remains crucial. Integration of digital health technologies and structured care pathways shows promise in improving patient outcomes.

## Graphical abstract

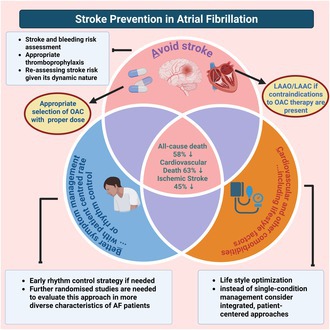

## 1 INTRODUCTION

The global prevalence of atrial fibrillation (AF), the most common arrhythmia worldwide, has been steadily increasing, with data from the Framingham Heart Study showing a three‐fold rise over the past five decades.^1^ Indeed, the Global Burden of Disease project reported 37.57 million prevalent cases of AF worldwide and 3.05 million new cases in 2017.^2^ In European countries, AF accounts for up to 2.6% of annual healthcare costs, primarily due to hospitalisations and complications like stroke, which alone cost the EU an estimated €45 billion in 2015.^3^, ^4^

AF is associated with a 4‐ to 5‐fold increased risk of stroke, and AF‐related strokes significantly increase the likelihood of long‐term disability or death.^5^, ^6^ The risk of stroke in AF patients varies and is influenced by several other risk factors, including age ≥ 65 years, hypertension, diabetes, previous stroke or transient ischaemic attack, vascular disease, heart failure and female sex.^7^, ^8^ Strokes in AF patients are associated with higher morbidity and mortality, with longer hospital stays, increased in‐hospital death rates and more frequent discharge to care facilities, while infarcts tend to be larger and more cortical, although the risk of silent infarcts is similar to non‐AF patients.^9^ While previous research has focused on the stroke risk associated with individual factors, it is now clear that the presence of multiple comorbidities in AF patients (that tend to cluster together) significantly increases the risk of stroke, thromboembolism and other adverse clinical complications.^10^, ^11^

Recent advances in technology, particularly the widespread adoption of wearable devices and continuous monitoring systems, have revolutionised AF detection and management, enabling earlier intervention with the potential to improve outcomes. However, significant gaps remain in clinical practice, including sub‐optimal anticoagulation rates, inadequate risk assessment and appropriate treatment and inconsistent application of integrated care approaches.

Stroke prevention in AF is more than oral anticoagulation (OAC) alone, as a continued residual risk of adverse clinical outcomes is evident despite anticoagulation.^12^ Hence, over the last decade guidelines have moved towards a holistic or integrated care approach to AF management; adherence to such an approach has demonstrated a reduction in mortality, stroke, bleeding and hospitalisations.^13^

Given recent advancements in stroke prevention strategies, there is a need for a review to synthesise current evidence, demonstrating the important challenges facing clinicians and highlighting evidence gaps requiring further research, leading to more appropriate and effective prevention, diagnosis and management of stroke in AF. The aim of this narrative review was therefore to offer an updated review of the approach to stroke prevention in AF, emphasising the importance of holistic or integrated care management, ongoing dynamic risk assessment and tailored stroke prevention strategies for specific AF patient groups.

Four major electronic databases (PubMed, Embase, Scopus and Web of Science) were searched for relevant guidelines, randomised controlled trials, systematic reviews, meta‐analyses and large observational studies on stroke prevention in atrial fibrillation, published through February 1, 2025.

## 2 IMPROVING STROKE PREVENTION BY IMPROVING DETECTION AND DIAGNOSIS OF ATRIAL FIBRILLATION

A diagnosis of AF can be confirmed using either a standard 12‐lead ECG recording or a single‐lead ECG tracing lasting at least 30 s, both of which must be reviewed by a physician.^14^

### 2.1 Digital health wearables

Digital health wearables, like smartwatches and fitness trackers, have proven highly effective in detecting AF through photoplethysmography (PPG) and electrocardiogram sensors.^15^ In the Huawei Heart Study, the feasibility and effectiveness of smart device–based PPG technology for AF screening were evaluated in a large‐scale Chinese population. Among over 187,000 individuals who used wearable devices to monitor their pulse rhythm, a .23% detection rate of suspected AF was observed; detection of AF increased with age. Follow‐up confirmed AF in 87.0% of suspected cases, demonstrating a high positive predictive value of 91.6%. Notably, 95.1% of identified AF cases were successfully integrated into a management programme, with approximately 80% of high‐risk patients receiving anticoagulation therapy. This study underscores the significant potential of mobile health technologies for early AF detection and management, offering an effective strategy to mitigate stroke risk and associated complications.^16^

Furthermore, other recent studies indicate that devices like the Apple Watch and Fitbit demonstrate positive predictive values surpassing 84% and 95%, respectively, highlighting their utility in early detection of AF, especially among asymptomatic individuals.^17^, ^18^ In a systematic review and meta‐analysis of AF detection (11 studies. *n* = 4241 participants), all included studies assessed the diagnostic performance of single‐lead ECG recordings from the Apple Watch, using the 12‐lead ECG as the reference standard. The pooled analysis demonstrated a sensitivity of 94.8% (95% CI: 91.7%–96.8%) and a specificity of 95.0% (95% CI: 88.6%–97.8%) for the Apple Watch ECG in detecting AF.^19^ Despite the excellent sensitivity and specificity of such devices, confirmation of AF by a physician is required to initiate appropriate treatment. Early AF detection through wearables allows for timely identification of AF and initiation of anticoagulation therapy, which can significantly reduce stroke risk.^20^ While wearables show great potential for early AF screening, more research and integration into standard care are needed to optimise patient outcomes.

### 2.2 Cardiac implantable electronic devices

Cardiac implantable electronic devices (CIEDs) can play an important role in the detection of sub‐clinical AF or atrial high rate episodes (AHREs), which are defined as atrial tachyarrhythmias (atrial rate ≥ 175/min) lasting more than 5 min.^21^ There is a growing body of evidence that AHREs are associated with progression to clinical AF and increased risk of stroke and thromboembolic events.^21^, ^22^, ^23^ Importantly, CIEDs can be used as an important AF detection tool, leading to the timely diagnosis of clinical AF, appropriate treatment, translating to improved outcomes.

### 2.3 Clinical risk scores

Several clinical risk scores, such as the C_2_HEST, mC_2_HEST, HAVOC and HATCH scores, have been developed to predict new‐onset AF. In a systematic review and meta‐analysis involving over 11 million patients, the C_2_HEST score demonstrated a pooled area under the curve (AUC) of .70 (95% CI: .66–.74).^24^ Additionally, a prospective longitudinal study of 4524 patients from diverse ethnic backgrounds reported that the mC_2_HEST score showed good predictive performance for AF at 10 years, with a C‐index of .72 (95% CI: .701–.753).^25^ Nevertheless, further studies are warranted to improve the predictive accuracy of these clinical risk scores, potentially by integrating advanced machine learning techniques.

### 2.4 Artificial intelligence detected atrial fibrillation

Artificial intelligence (AI) can play an important role in detecting AF, with recent studies demonstrating considerable accuracy.^26^, ^27^ In a nonrandomised interventional trial of 1003 patients without prior diagnosis of AF but with at least one stroke risk factor, AI‐guided screening was associated with significantly higher AF detection compared to usual care (high‐risk group: 3·6% [95% CI 2.3–5.4] with usual care vs. 10·6% [8.3–13.2] with AI‐guided screening, *p* < .0001; low‐risk group: .9% vs. 2·4%, *p* = .12).^26^ In a retrospective longitudinal study, 180,922 patients (126,526 patients in the training dataset, 18,116 patients in the internal validation dataset, and 36,280 patients in the testing dataset) with normal sinus rhythm ECG were analysed. In the test dataset, 8.4% (*n* = 51) of the patients had already been diagnosed with AF before the ECG was used in the model while they were in normal sinus rhythm. AI‐enabled ECG identified the presence of AF with a high predictive performance (AUC .87, 95% confidence interval (CI) .86–.88; sensitivity 79.0%, 77.5–80.4, specificity 79.5%, 79.0–79.9; F1 score, 39.2% 38.1–40.3).^27^

Despite rapid progress in AI over the years, it has some limitations, notably that it operates as a black box meaning that it does not display how it behaves or how it reaches its decisions. Therefore, it is of great importance to find reasonable explanations for AI predictions to maximise its benefits in clinical contexts.

### 2.5 Atrial fibrillation (AF) screening

AF screening can help identifying asymptomatic patients with AF who are at risk of stroke. In a meta‐analysis of four randomised trials with a total of 35,836 participants, it was reported that AF screening was associated with a decreased likelihood of developing stroke (relative risk .91; 95% confidence interval: .84–.99).^28^ The ongoing SAFER trial is recruiting around 82,000 individuals aged more than 70 years without OAC from general practices in England. Following the SAFER pilot, this trial aims to determine whether population screening for AF reduces stroke risk.^29^

## 3 THE 4S‐AF SCHEME: A COMPREHENSIVE APPROACH TO CHARACTERISATION OF ATRIAL FIBRILLATION

The 4S‐AF framework has four domains: stroke risk (St), symptoms severity (Sy), severity of AF burden (Sb) and substrate (Su). Stroke risk assessment using the CHA_2_DS_2_‐VASc score; evaluation of symptom severity with the European Heart Rhythm Association (EHRA) score; assessment of AF burden; and analysis of underlying substrate factors such as age, structural heart disease and comorbidities.^30^ The 4S‐AF framework has facilitated a holistic understanding of the individual patient's AF profile leading to tailored AF management.

**FIGURE 1:**
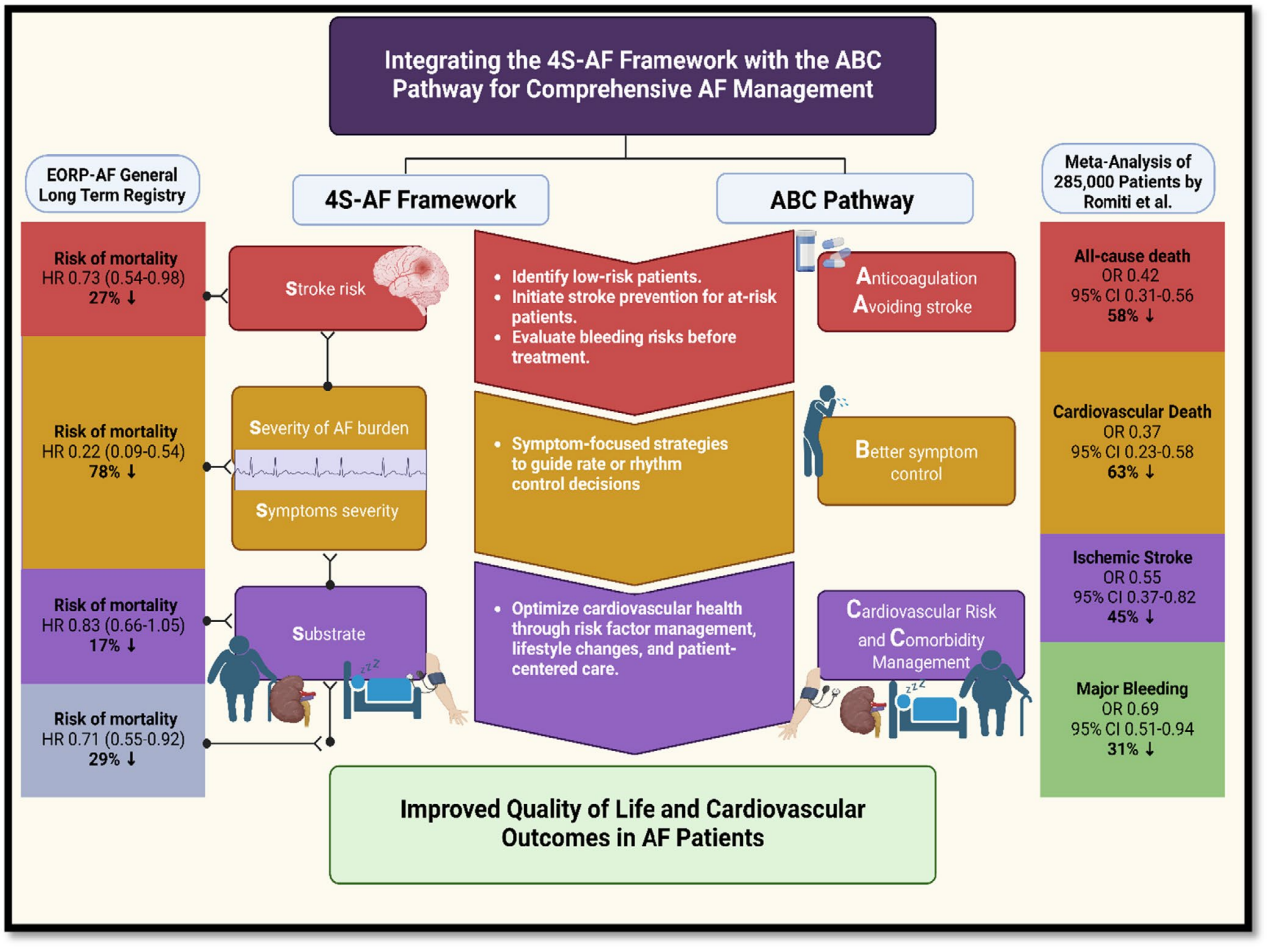
Integrated model of 4S‐AF framework and ABC pathway for AF management. Schematic representation showing the interrelationship between 4S‐AF domains and ABC pathway components for optimising patient care in atrial fibrillation.^13^, ^140^

Recent studies have demonstrated that not only is it feasible to characterise and manage AF patients using the innovative 4S‐AF scheme, but also that utilising these tools is linked to improved clinical outcomes.^30^, ^31^, ^32^, ^33^, ^34^ Table 1 summarises studies evaluating the use of the 4S‐scheme on the clinical outcome of AF patients. Figure 1 demonstrates the integration of the 4S‐AF framework with the Atrial Fibrillation Better Care (ABC) pathway, illustrating a comprehensive approach to AF management.

**TABLE 1:** Studies assessing the impact of the 4S scheme on clinical outcomes in atrial fibrillation patients.

First author, publication year	Sample size	Study design	Main finding
Malavasi, 2021^32^	615	Prospective longitudinal	Deviation from rate control recommendation: No significant impact on outcomes when deviating from the 4S‐AF score's recommendation for rate control. Nonadherence to rhythm control recommendation: Associated with significantly higher risks: ○ All‐cause mortality: HR 7.59 (95% CI 1.65–35.01). ○ Composite outcomes: HR 2.69 (95% CI 1.19–6.06).
Ding, 2022^140^	6321	Prospective longitudinal	Addressing all 4S‐AF domains: Independently reduced the risk of all‐cause mortality: aHR .71 (95% CI: .55–.92). Failure to treat any 4S‐AF domain: Significantly increased the risk of all‐cause mortality: aHR 1.35 (95% CI: 1.16–1.56).
Rivera‐Caravaca, 2022^33^	1479	Prospective longitudinal	Predictive performance of the 4S‐AF scheme for management strategy: Continuous form: C‐index = .77 (95% CI: .75–.80). Categorical form: C‐index = .75 (95% CI: .72–.78). Risks for patients in the ‘red category’: All‐cause mortality: aHR 1.75 (95% CI: 1.02–2.99). Composite outcomes: aHR 1.60 (95% CI: 1.05–2.44).
Chao, 2022^34^	4666	Prospective longitudinal	Risk of adverse events by 4S‐AF score tertiles: Second tertile (4–5 points): 2.5‐fold increase compared to the first tertile (OR 2.478, 95% CI: 1.678–3.661, *p* < .001). Third tertile (6–9 points): 3.5‐fold increase compared to the first tertile (OR 3.484, 95% CI: 2.322–5.226, *p* < .001). Consistency across countries: Results were consistent across five countries (*p* for interaction > .05). Benefit of treating all 4S‐AF domains: Linked to a reduced risk of composite outcomes (aOR .384, *p* < .001). No significant variation between countries (*p* for interaction = .234).
Guo, 2022^30^	6419	Prospective longitudinal	Patient groups based on 4S‐AF and ABC pathway use: Group 1: 59.8% (3503 patients) were neither assessed with the 4S‐AF scheme nor managed with the ABC pathway. Group 2: 28.0% (1795 patients) were either assessed by the 4S‐AF scheme or ABC‐adherent, but not both. Group 3: 17.4% (1121 patients) were assessed with the 4S‐AF scheme and managed with the ABC pathway. Composite outcome (all‐cause mortality and thromboembolic events): Group 3 showed the greatest benefit (OR .19; 95% CI .12–.31). Group 2 also showed significant benefit (OR .28; 95% CI .20–.37). All‐cause mortality alone: Group 2: OR .18 (95% CI .12–.27). Group 3: OR .14 (95% CI .07–.25).

Abbreviations: 4S‐AF, stroke risk, symptoms, severity, and substrate in atrial fibrillation scheme; ABC, Atrial Fibrillation Better Care; aHR, adjusted hazard ratio; aOR, adjusted odds ratio; CI, confidence interval; C‐index, concordance index (a measure of predictive accuracy); HR, hazard ratio; MI, myocardial infarction; OR, odds ratio.

## 4 COMPREHENSIVE MANAGEMENT OF ATRIAL FIBRILLATION

**FIGURE 2:**
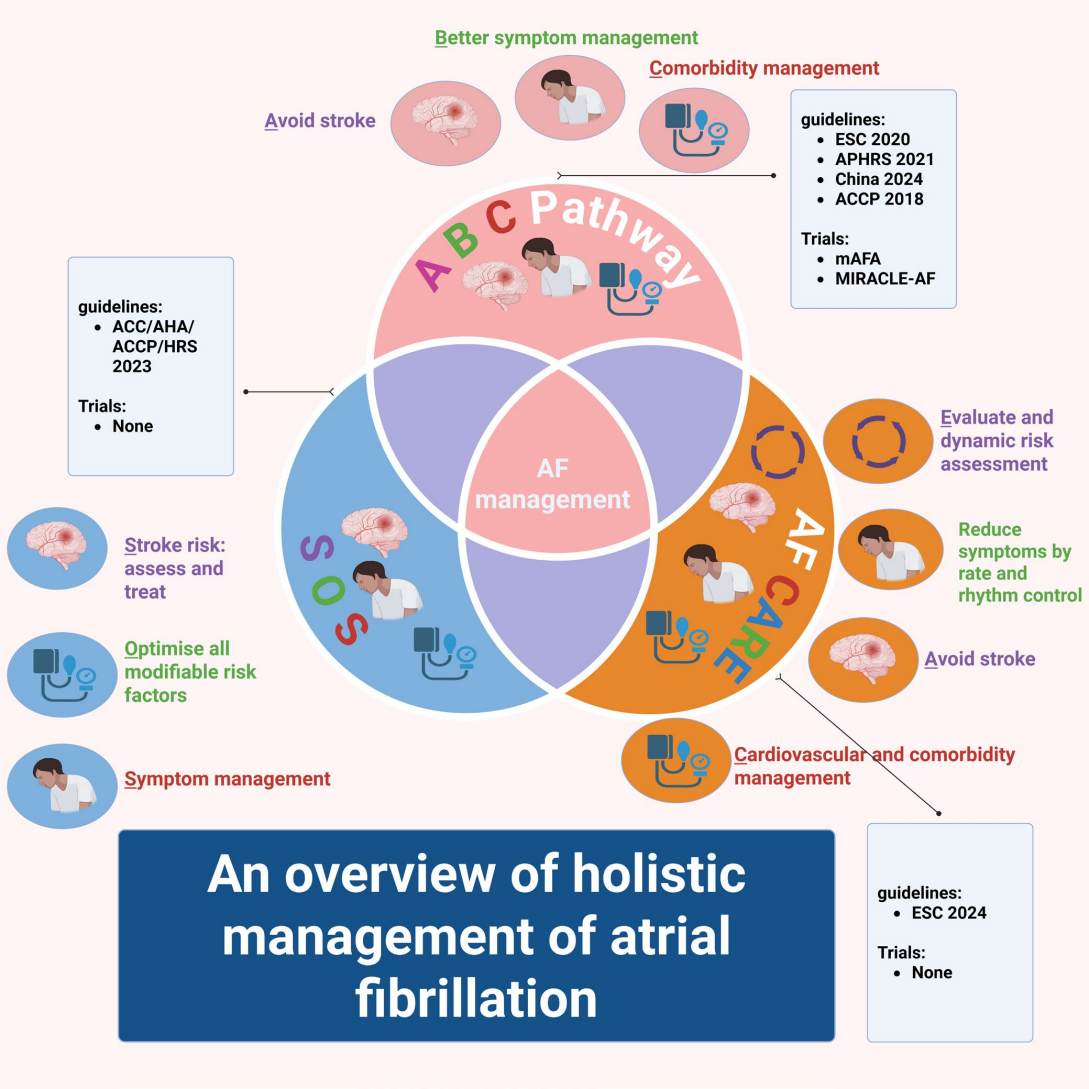
Clinical acronyms and decision‐making tools in contemporary AF management. mAFA: Mobile Health Technology for Improved Screening and Optimised Integrated Care in AF; MIRACLE‐AF: A novel Model of IntegRAted Care of oLdEr patients with Atrial Fibrillation in rural China.

A comprehensive approach to manage risk in patients with AF includes several key steps, which can be summarised using the evidence‐based ABC pathway: (A) Anticoagulation to prevent stroke/thromboembolism, (B) Patient‐centred decisions on rate or rhythm management for better symptom control and (C) Managing cardiovascular risk factors, comorbidities and lifestyle changes.^35^ The ABC pathway is supported by clinical trials and real‐world evidences.^36^, ^37^, ^38^, ^39^, ^40^, ^41^, ^42^ Table 2 summarises key studies evaluating the impact of ABC pathway on patient outcomes. Variations of the ‘ABC’ acronym (which are untested in clinical trials^43^) have been used in the recent US guidelines (as ‘SOS’, i.e. stroke, other comorbidities, rate or rhythm (Symptom) control)^44^ and the 2024 European guidelines^45^ (as ‘CARE’, i.e., comorbidities, avoid stroke, rate or rhythm control, evaluation) (Figure 2).

**TABLE 2:** Impact of the ABC pathway strategy on outcomes in atrial fibrillation patients.

First author, publication year	Sample size	Study design	Main finding
Ritchie, 2024^141^	14,493	Retrospective cohort	Composite outcome: No significant association observed: aHR 1.01 (95% CI, .97–1.05). Specific health outcomes: Reduced risk of myocardial infarction: aHR .70 (95% CI, .50–.98). Increased risk of hemorrhagic stroke: aHR 1.59 (95% CI, 1.06–2.39). Other individual outcomes: No significant associations identified.
Haghjoo, 2024^142^	1341	Prospective longitudinal study	Reductions in adverse outcomes with ABC pathway: Stroke/TIA: Decreased from 6.3% to 2.2% (*p* = .002). Systemic thromboembolism: Reduced from 1.4% to .0% (*p* = .04). Nosebleeds: Reduced from .8% to .6% (*p* = .04). Skin bruising: Dropped from 1.2% to .0% (*p* = .002). Heart failure: Declined from 7.7% to 4.7% (*p* = .04). Symptom improvement: Proportion of patients in EHRA Class I‐II increased from 93.3% at enrolment to 98.1% at follow‐up.
Rivera‐Caravaca, 2023^143^	1045	Prospective cohort	Lower event Rates with ABC Pathway Adherence During Follow‐up All‐cause mortality: 6.56 vs. 13.76 (*p* < .001) Noncardiovascular outcomes: 11.94 vs. 19.65 (*p* < .001) Major adverse cardiovascular events (MACE): 7.75 vs. 11.88 (*p* = .006)
Romiti, 2023^144^	24,608	Prospective longitudinal study	Primary outcome: Adherence to the ABC pathway significantly reduced the risk, particularly in fully adherent patients: aHR .54 (95% CI, .44–.67; *p* < .0001). Specific risk reductions: Mortality: aHR .89 (95% CI, .79–1.00; *p* = .048). Thromboembolism: aHR .78 (95% CI, .65–.94; *p* = .0078). Major Adverse Cardiac Events (MACE): aHR .82 (95% CI, .71–.95; *p* = .0071).
Patel, 2022^145^	20,926	Post hoc analysis of ENGAGE AF‐TIMI 48 trial	Adverse outcome reductions: Stroke/systemic embolic events: HR .54 (95% CI: .47–.63). Major bleeding: HR .66 (95% CI: .58–.75). Major adverse cardiac events: HR .53 (95% CI: .48–.58). Primary net clinical outcome: Reduced risk, HR .61 (95% CI: .56–.65). Cardiovascular outcomes: Hospitalisation: HR .78 (95% CI: .74–.83). Cardiovascular death: HR .52 (95% CI: .46–.58). All‐cause mortality: Reduced risk, HR .56 (95% CI: .51–.62). All comparisons were statistically significant (*p* < .001).
Guo, 2022^37^	3520	Prospective longitudinal study	Lower incidence of primary outcome with ABC pathway adherence After 1 year, patients managed with the ABC pathway had a significantly lower incidence of the primary composite outcome (all‐cause death or thromboembolic events) compared to nonadherent patients (1.5% vs. 3.6%; *p* < .01) Multivariate analysis of ABC pathway adherence and risk reduction ABC adherence was independently associated with a reduced risk of the primary outcome (OR: .51; 95% CI: .31–.84)
Ding, 2022^146^	6646	Prospective longitudinal study	Multivariable analysis of risk reduction with ABC pathway adherence Reduced risks for high‐risk patients (aHR .64; 95% CI, .51–.80) Reduced risks for patients with chronic kidney disease (aHR .51; 95% CI, .37–.70) Reduced risks for elderly patients (aHR .69; 95% CI, .53–.90)
Gumprecht, 2020^147^	2021	Prospective longitudinal study	Composite outcome (ischaemic stroke or systemic embolism, all‐cause death and cardiovascular hospitalisation) rates: ABC care group: 20.8%. Non‐ABC group: 29.3%. *p*‐value: .02 (significant). Mortality rates: ABC care group: 7.3%. Non‐ABC group: 13.1%. *p*‐value: .033 (significant). Adjusted risk reduction (multivariable analysis): Composite outcome: HR .53 (95% CI, .36–.8; *p* = .002). Mortality: HR .46 (95% CI, .25–.86; *p* = .015).
Yoon, 2019^49^	204,842	Prospective cohort	Mortality rates: ABC‐compliant patients: .80 per 100 person‐years. Noncompliant patients: 2.72 per 100 person‐years. *p*‐value: <.001 (significant). Composite outcome rates (death, ischaemic stroke, major bleeding and myocardial infarction): ABC‐compliant patients: 2.34 per 100 person‐years. Noncompliant patients: 5.92 per 100 person‐years. *p*‐value: <.001 (significant). Adjusted risks (adjusted Cox regression): All‐cause death: aHR .82 (95% CI, .78–.86). Composite outcome: aHR .86 (95% CI, .83–.89).
Pastori, 2018^148^	907	Prospective cohort	Risk reduction: Lower annual risk of cardiovascular events: Optimally managed ABC group: 1.8% (95% CI, .9–3.0). Suboptimal ABC group: 4.5% (95% CI, 3.7–5.5). Difference between groups was significant (*p* = .001). Hazard ratio: Cardiovascular events in the optimally managed group: HR .40 (95% CI, .22–.74, *p* = .003).

Abbreviations: ABC, Atrial Fibrillation Better Care; AF, atrial fibrillation; aHR, adjusted hazard ratio; CI, confidence interval; HR, hazard ratio; IQR, interquartile range; IRR, incidence rate ratio; MACE, major adverse cardiovascular events; OR, odds ratio; TIA, transient ischaemic attack; VKA, vitamin K antagonist.

The mAFA trial, a cluster randomised study in China, enrolled 3324 AF patients (≥18 years) across 40 cities, comparing ABC pathway care supported by a mobile application (mAFA) intervention (*n* = 1646, mean age 67) to usual care (*n* = 1678, mean age 70). Over ~9 months of follow‐up, the mAFA group had significantly lower rates of the composite endpoint of ischaemic stroke, systemic thromboembolism, death and rehospitalisation (1.9% vs. 6.0%; HR: .39; 95% CI: .22–.67; *p* < .001), as well as reduced rehospitalisation rates (1.2% vs. 4.5%; HR: .32; 95% CI: .17–.60; *p* < .001) compared to those receiving usual care.^4^

The MIRACLE‐AF trial was a cluster randomised clinical trial (RCT) which investigated a telemedicine‐based, village doctor‐led integrated care model based on the ABC pathway compared to usual care.^36^ Over 34.0 months follow‐up, there was improved adherence to all ABC criteria for those receiving integrated AF care compared to usual care (*p* < .001).^46^ The rate of the composite cardiovascular event outcome was lower in the intervention group than with usual care (6.2% vs. 9.6% per year; hazard ratio (HR), .64 (95% CI, .50–.82); *p* < .001).

A comprehensive meta‐analysis (eight studies, *n* = 285,000 patients) demonstrated that adherence to the ABC pathway was significantly associated with lower adverse outcomes compared to usual care. The findings revealed a notable reduction in all‐cause mortality by 58%, cardiovascular death by 63%, stroke incidence by 45% and major bleeding events by 31%.^13^ Consistently across various studies including a post‐hoc analysis of Atrial Fibrillation Follow‐Up Investigation of Rhythm Management (AFFIRM) trial,^47^ retrospective analysis from Chinese AF registry,^48^ and a nationwide prospective cohort study,^49^ adherence to the ABC pathway has consistently shown benefits in reducing mortality, cardiovascular events and healthcare costs in patients with AF.

Although ABC adherence is also associated with improvements in clinically complex AF patients, more specific strategies such as more personalised risk‐based anticoagulation decisions, optimisation of comorbidity management and enhanced digital health interventions are needed in this high‐risk subset of AF patients.^50^ Further larger randomised trials investigating the role of the ABC pathway are required, enrolling patients from different ethnic groups and across more diverse health care settings (e.g. rural and underserved areas, tertiary academic centres and telemedicine‐based care environments). The ABC pathway is being investigated in European countries as part of a clinical trial in the AFFIRMO programme.^51^

### 4.1 Stroke prevention through effective anticoagulation

Effective anticoagulation with appropriate OAC therapy (vitamin K antagonists (VKAs) or direct oral anticoagulants (DOAC)) in patients with AF is a key strategy for preventing stroke and systemic embolism. The standard approach for assessing stroke risk is the comprehensive CHA_2_DS_2_‐VASc score.^52^, ^53^ However, there are several important considerations and clarifications needed regarding its application and effective implementation in clinical practice. Importantly, growing evidence suggests that stroke risk is not fixed but dynamic, fluctuating with age, the development of new comorbidities and changes influenced by polypharmacy and management of risk factors. Some studies indicate that the baseline CHA_2_DS_2_‐VASc score may underestimate stroke risk, as it fails to consider the addition of incident risk factors (like hypertension or diabetes) over time. This concept was first proposed by Chao et al. who analysed 31,039 AF patients and showed that stroke risk, as measured by the CHA_2_DS_2_‐VASc score, is dynamic. The score increased over time, with 64.4% of stroke patients developing new comorbidities during a mean follow‐up of 3.4 ± 3.7 years. The change in CHA_2_DS_2_‐VASc score (AUC .74, 95% CI .73–.75), reflecting changes in risk, was a stronger predictor of ischaemic stroke than baseline (AUC .57, 95% CI .56–.58) or follow‐up (AUC .72, 95% CI .72–.73) CHA_2_DS_2_‐VASc scores, emphasising the need for regular risk reassessment of stroke risk in AF management.^54^ In a French prospective cohort of 608,108 patients, changes in the CHA_2_DS_2_‐VASc score (AUC = .63, 95% CI .633–.640) over a mean follow‐up of 2.2 ± 2.4 years were found to be more accurate in predicting ischaemic stroke than baseline CHA_2_DS_2_‐VASc score (AUC .61, 95% CI .60–.61). The risk of stroke increased as patients aged or acquired additional comorbidities.^55^

The frequency of reassessing stroke risk varies by international guideline. The 2020 ESC guidelines recommend reassessing stroke risk in AF patients 4–6 months after the initial evaluation to adjust anticoagulation as needed.^56^ In contrast, the 2021 Asia Pacific Heart Rhythm Society (APHRS) guidelines advise a more frequent reassessment, at least annually, and ideally every 4 months, due to the dynamic nature of stroke risk influenced by aging and new comorbidities, which ensures timely updates to anticoagulation therapy for optimal stroke prevention.^57^ The 2024 ESC guidelines recommend periodic individualised reassessment of thromboembolic risk in AF patients to ensure that anticoagulation is initiated when clinically appropriate.^45^, ^58^ In summary, given the dynamic nature of stroke risk influenced by aging and the development of new comorbidities, reassessment of the CHA_2_DS_2_‐VASc score every 4–6 months is crucial for optimising anticoagulation therapy and improving stroke prevention outcomes in AF patients.

#### 4.1.1 Assessing the role of gender in AF stroke risk: CHA_2_ DS_2_‐VASC versus CHA_2_ DS_2_‐VA scores

The 2024 ESC guidelines proposed using the nonsex CHA_2_DS_2_‐VASc (i.e. CHA_2_DS_2_‐VA) score, which excludes birth sex or gender criteria. This recommendation, based on expert consensus (Level of Evidence C), reflects concerns that including sex into risk stratification can complicate clinical decision‐making and may not adequately account for individuals who are nonbinary, transgender or undergoing gender‐affirming hormone therapy.^45^ However, sex is an important consideration in the management of stroke risk for AF patients. Women with AF face a greater risk of thromboembolic stroke than men, even after accounting for other stroke risk factors. This heightened risk, as well as the under‐treatment with OAC of women with AF, was one reason why female sex is included as a factor in the CHA_2_DS_2_‐VASc score for assessing stroke risk.^59^, ^60^ In addition, women are at an increased risk of stroke due to factors specific to them, such as hypertensive disorders during pregnancy, menopause and the use of hormone replacement therapy.^61^ Women also face an increased lifetime risk of stroke and often experience worse outcomes after a stroke, which together contribute to a higher overall stroke burden in females compared to males.^62^

Several studies have explored the use of the CHA_2_DS_2_‐VA score for stroke risk stratification in AF patients. Nevertheless, the early studies faced several methodological shortcomings, and one study was subsequently retracted.^63^, ^64^ For instance, a sub‐analysis of the J‐RHYTHM Registry in Japanese patients found that the CHA_2_DS_2_‐VA score performed comparably to the traditional CHA_2_DS_2_‐VASc score in predicting thromboembolic events, showing no significant loss in predictive power while simplifying the risk assessment.^63^ In addition, these risk scores were based solely on baseline risk factors, neglecting dynamic changes over time and focused exclusively on patients who were not on anticoagulation therapy at baseline, without considering the initiation of OAC during follow‐up. A UK study examining the impact of female sex in AF patients found that women had a lower adjusted rate of adverse events compared to men (HR .89, 95% CI .87–.92; *p* < .001), primarily due to lower mortality (HR .86, 95% CI .83–.89; *p* < .001), while no significant differences were observed for ischaemic stroke, arterial thromboembolism or vascular dementia (adjusted HR 1.00, 95% CI .94–1.07; *p*‐value = .87). The study also showed that CHA_2_DS_2_‐VA (excluding sex) performed slightly better than CHA_2_DS_2_‐VASc for predicting primary outcomes, but since the study excluded AF patients aged ≥75 years and those with prior stroke, it was effectively comparing CHD‐VASc and CHD‐VA rather than CHA_2_DS_2_‐VASc and CHA_2_DS_2_‐VA per se.^65^ In contrast, some studies suggest that eliminating the female sex criterion from the score led to only slight improvements in the c‐index and may risk underestimating stroke risk in specific populations.^66^, ^67^ Nonetheless, the adoption of CHA_2_DS_2_‐VA and the removal of the sex criterion from the stroke risk assessment model has been subject to some debate.^43^

#### 4.1.2 Anticoagulation therapies and alternative interventions for stroke prevention in AF

**FIGURE 3:**
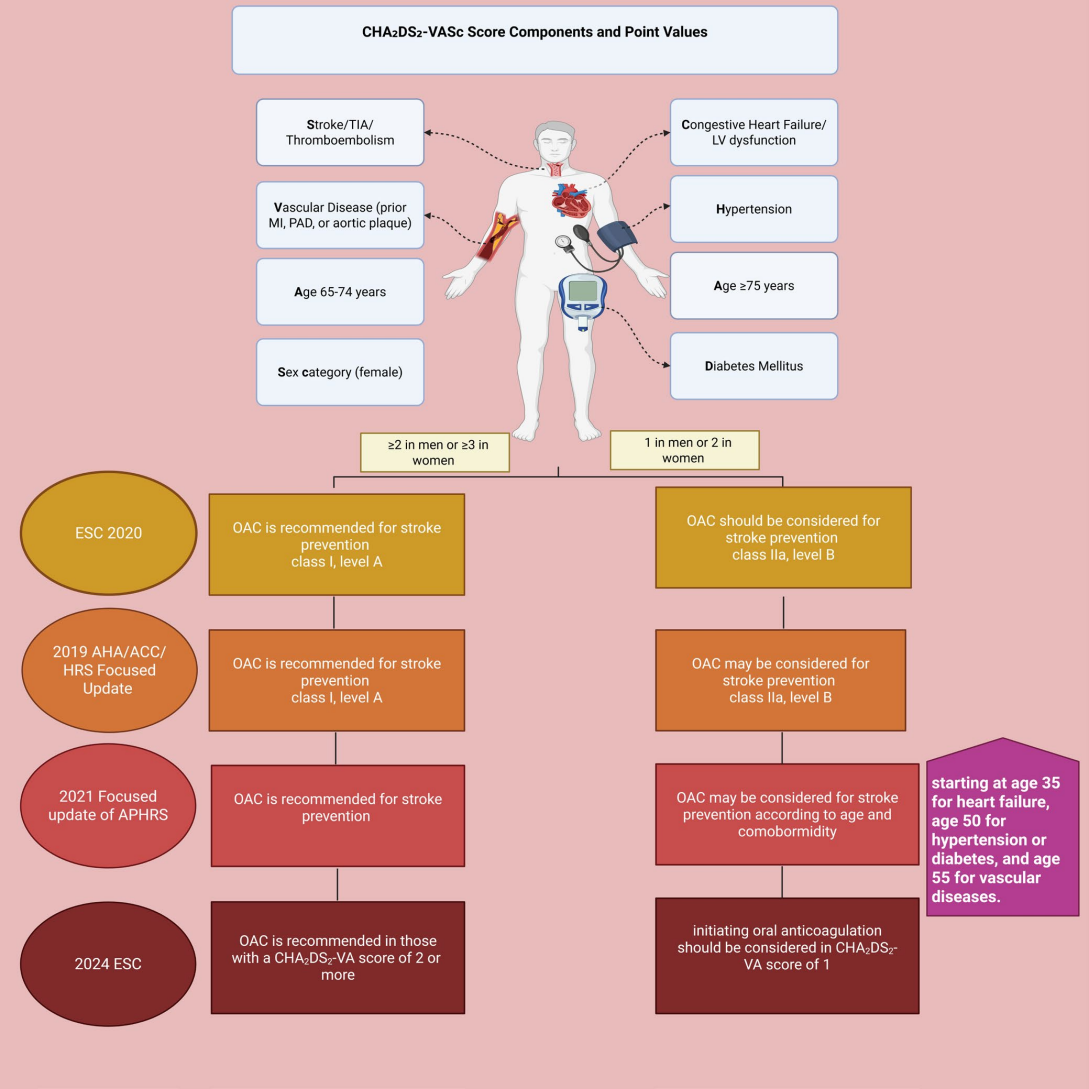
Integration of CHA_2_DS_2_‐VASc Score Calculation with Contemporary Guideline Recommendations for Anticoagulation. A comprehensive overview of stroke risk factor scoring and corresponding recommendations for anticoagulation initiation from major international guidelines (ESC 2024,^14^ AHA/ACC/HRS 2019^149^ and APHRS 2021^150^), stratified by sex‐specific CHA_2_DS_2_‐VASc scores. The CHA_2_DS_2_‐VASc score components include congestive heart failure or left ventricular dysfunction (1 point), hypertension or history of high blood pressure (1 point), age ≥ 75 years (2 points), diabetes mellitus (1 point), stroke, TIA or thromboembolism (2 points), vascular disease including prior myocardial infarction, peripheral artery disease or aortic plaque (1 point), age 65–74 years (1 point) and female sex category (1 point), with a maximum possible score of 9 points, where higher scores indicate a greater risk of stroke.

OAC therapy is a critical component in the prevention of stroke and systemic embolism, particularly in patients with AF.^68^ By reducing the risk of clot formation, OACs significantly lower the chances of stroke. Most guidelines for stroke prevention in AF patients recommend prescription of OAC, with a preference for a DOAC for all patients unless they are considered low risk for stroke (CHA_2_DS_2_‐VASc score of 0 for men or 1 for women), have a mechanical heart valve or moderate‐to‐severe mitral stenosis, or end stage renal disease. Table 3 and Figure 3 summarise the latest recommendations of recent guidelines for initiation of OAC therapy in AF patients for stroke prevention. Certain aspects of AF management in stroke patients, such as stroke occurring despite anticoagulation which presents diagnostic and clinical challenges, are beyond the scope of this narrative review.

**TABLE 3:** Recommendations in recent guidelines for starting anticoagulant therapy in AF patients for stroke prevention.

Guideline	Recommendation for high thromboembolic risk patients	Recommendation intermediate thromboembolic risk patients
2024 ESC Guidelines^14^	OAC is recommended in those with a CHA_2_DS_2_‐VA score of 2 or more and should be considered in those with a CHA_2_DS_2_‐VA score of 1, following a patient‐centered and shared care approach.	A CHA_2_DS_2_‐VA score of 1 should be considered an indicator of elevated thromboembolic risk for decisions on initiating oral anticoagulation (class IIa, level = C).
2020 ESC Guidelines^56^	OAC is recommended for stroke prevention in AF patients with CHA_2_DS_2_‐VASc score > _2 in men or >_3 in women (class I, level A). OAC should be considered for stroke prevention in AF patients with a CHA_2_DS_2_‐VASc score of 1 in men or 2 in women (class IIa, level B).	OAC should be considered for stroke prevention in AF patients with a CHA_2_DS_2_‐VASc score of 1 in men or 2 in women (class IIa, level B).
2019 AHA/ACC/HRS Focused Update of the 2014 AHA/ ACC/HRS Guideline^149^	For patients with AF and an elevated CHA_2_DS_2_‐VASc score of 2 or greater in men or 3 or greater in women, oral anticoagulants are recommended.	For patients with AF (except with moderate to‐severe mitral stenosis or a mechanical heart valve) and a CHA_2_DS_2_‐VASc score of 1 in men and 2 in women, prescribing an oral anticoagulant to reduce thromboembolic stroke risk may be considered (COR = 1, LOE = A).
2021 Focused update of the 2017 APHRS guideline^150^	In patients with AF with CHA_2_DS_2_‐VASc score ≥2 in men or ≥3 in women, OAC is recommended for stroke prevention.	In patients with atrial fibrillation (AF) who have a CHA_2_DS_2_‐VASc score of 1 for men or 2 for women, the use of oral anticoagulants (OAC) should be considered for stroke prevention. The decision to use direct oral anticoagulants (DOACs) can be further refined by applying specific age thresholds based on comorbidities: for instance, starting at age 35 for heart failure, age 50 for hypertension or diabetes, and age 55 for vascular diseases.
2023 ACC/AHA/ACCP/HRS guideline^44^	For patients with AF and an estimated annual thromboembolic risk of ≥2% per year (e.g. CHA_2_DS_2_‐VASc score of ≥2 in men and ≥3 in women), anticoagulation is recommended to prevent stroke and systemic thromboembolism.	For patients with AF and an estimated annual thromboembolic risk of ≥1% but <2% per year (equivalent to CHA_2_DS_2_‐VASc score of 1 in men and 2 in women), anticoagulation is reasonable to prevent stroke and systemic thromboembolism.
2024 Chinese Expert Consensus Guidelines^58^	In patients at intermediate risk for stroke (i.e. stroke prevalence of 1%–2%/year, that is, CHA_2_DS_2_‐VASc =1 in male or = 2 in female) could benefit from the modification of risk factors.	For the patients with intermediate‐ or high‐risk stroke, but with long‐term oral anticoagulant contraindications due to irreversible causes, percutaneous left atrial appendage closure is recommended.
2020 Canadian Cardiovascular Society/Canadian Heart Rhythm Society Comprehensive Guidelines^151^	We recommend that all patients with AF should undergo annual assessment of their risk of stroke/ systemic embolism, irrespective of their clinical pattern of AF.	We recommend that patients who present with AF in the acute care setting have their need for long‐term antithrombotic therapy be determined using the CCS Algorithm (CHADS‐65)
Australian Clinical Guidelines^152^	Oral anticoagulation therapy to prevent stroke and systemic embolism should be considered in patients with nonvalvular AF whose CHA_2_DS_2_‐VA score is 1.	Oral anticoagulation therapy to prevent stroke and systemic embolism is recommended in patients with nonvalvular AF whose CHA_2_DS_2_‐VA score is 2 or more, unless there are contraindications to anticoagulation

Abbreviations: ABC, Atrial Fibrillation Better Care; ACC, American College of Cardiology; AF, atrial fibrillation; AHA, American Heart Association; APHRS, Asia Pacific Heart Rhythm Society; CHA_2_DS_2_‐VA, congestive heart failure, hypertension, age ≥ 75 years, diabetes, stroke/tia/thromboembolism, vascular disease, age; CHA_2_DS_2_‐VASc, congestive heart failure, hypertension, age ≥ 75 years, diabetes, stroke/tia/thromboembolism, vascular disease, age 65–74 years, sex category; COR, class of recommendation; DOACs, direct oral anticoagulants; EACTS, European Association of Cardio‐Thoracic Surgery; ESC, European Society of Cardiology; HRS, Heart Rhythm Society; LOE, level of evidence; OAC, oral anticoagulants.

##### Vitamin K antagonists

VKAs exert their anticoagulant effect indirectly by inhibiting the vitamin K epoxide reductase complex subunit 1. This inhibition disrupts the activation of vitamin K‐dependent coagulation factors (II, VII, IX and X), as well as anticoagulant proteins (C, S and Z), leading to reduced blood clotting ability.^69^ The delay between taking the drug and achieving its full pharmacological effect typically ranges from 3 to 7 days. This period is necessary for the depletion or exhaustion of active coagulation factors. However, prothrombin time can increase more rapidly due to the quick inhibition of short‐lived coagulation factors like factor VII. The dosage of VKAs must be tailored individually due to variations in pharmacokinetics and pharmacodynamics among patients. This is determined by measuring the International Normalised Ratio (INR), which is calculated as the ratio of a patient's prothrombin time to that of a healthy individual. For patients with AF, the target INR should be maintained between 2.0 and 3.0 to ensure effective anticoagulation while minimising bleeding risk.^70^

##### Importance of time in therapeutic range (TTR)

The quality of anticoagulation control, as measured by time in therapeutic range (TTR), plays a crucial role in optimising the effectiveness and safety of VKA therapy in patients with AF. There is a significant relationship between increasing TTR (>65%) and reduced incidence of ischaemic stroke and intracranial haemorrhage (ICH).^71^ In a prospective cohort study of 8754 Chinese patients with AF, stroke rates progressively decreased from 7.34% per year in the lowest TTR quartile (<17.9%) to 3.10% per year in the highest quartile (>56.2%), while ICH incidence declined from 1.37% per year in the lowest TTR quartile to .74% per year in the highest quartile.^72^ While VKA efficacy improved with higher TTR, dabigatran demonstrated superior outcomes, with the lowest stroke (2.24% per year) and ICH (.32% per year) rates across all therapies.^72^ Hence, this study highlights the importance of maintaining high TTR levels (≥65%) to maximise the benefits of warfarin while emphasising the potential advantages of DOACs in stroke prevention and safety. In patients treated with VKAs or DOACs, it has been recommended to avoid NSAIDs, antifungals like fluconazole and voriconazole, and antidepressants like fluoxetine due to increased bleeding risk.^73^ Current evidence does not recommend dietary modifications when starting VKA therapy.^74^, ^75^

##### Evidence for warfarin and DOACs for stroke prevention in AF

DOACs work by directly inhibiting either Factor II (dabigatran) or Factor Xa (rivaroxaban, apixaban and edoxaban) and have several key benefits compared to VKAs, including stable anticoagulation effects, rapid onset action, fewer drug interactions and fixed dosing without the need for regular monitoring (for INR) or dietary limitations.^76^

The advent of DOACs (apixaban, dabigatran, edoxaban and rivaroxban) represented a paradigm shift in AF management. Both clinical trial and real‐world data have consistently demonstrated their comparable or superior efficacy and safety profile compared to warfarin.^77^, ^78^, ^79^ A meta‐analysis of 71,683 participants from the four DOAC RCTs (RE‐LY, ROCKET AF, ARISTOTLE and ENGAGE AF–TIMI 48) showed that DOACs were more effective and safer for several clinical outcomes compared to warfarin.^77^ DOACs significantly reduced stroke or systemic embolic events by 19% (risk ratio(RR) .81; 95% CI .73–.91; *p* < .0001), largely due to a significant reduction in haemorrhagic stroke (RR .49; 95% CI .38–.64; *p* < .0001). They were also associated with a lower risk of all‐cause mortality (RR .90; 95% CI .85–.95 *p* = .0003) and ICH (RR .48; 95% CI .39–.59, *p* < .0001) but increased gastrointestinal bleeding (RR 1.25; 95% CI 1.01–1.55; *p* = .04). The benefit in major bleeding was greater when TTR (with warfarin) was poor (below 66%). Low‐dose DOACs showed similar efficacy to warfarin for stroke prevention but had a better bleeding profile, albeit with a slightly higher risk of ischaemic strokes.^77^

A patient‐level network meta‐analysis of the four pivotal RCTs comparing DOACs to warfarin in patients with AF also found that standard‐dose DOACs significantly reduced the risk of stroke or systemic embolism (3.01% vs. 3.69%; HR .81, 95% CI .74–.89), death (7.76% vs. 8.42%; HR .92, 95% CI .87–.97) and intracranial bleeding (.63% vs. 1.40%; HR .45, 95% CI .37–.56), with no significant difference in major bleeding risk (5.05% vs. 5.94%; HR .86, 95% CI .74–1.01).^80^ Lower‐dose DOACs showed similar rates of stroke or embolism (3.96% vs. 3.69%; HR 1.06, 95% CI .95–1.19) but reduced risk of intracranial bleeding (.42% vs. 1.40%; HR .28, 95% CI .21–.37), death (8.29% vs. 8.42%; HR .90, 95% CI .83–.97) and major bleeding (4.34% vs. 5.94%; HR .63, 95% CI .45–.88).^80^

In a ‘real‐world’ meta‐analysis (21 studies, 605,771 patients) comparing DOACs demonstrated that both apixaban and dabigatran were associated with lower risk of major bleeding and gastrointestinal bleeding compared to rivaroxaban [(HR 2.0, 95% CI 1.6–2.5 and HR 1.2, 95% CI 1.0–1.5), respectively].^81^ These findings highlight differences in safety outcomes differences among outcomes that should be considered in management of AF patients, with choice of (D)OAC made on a patient‐by‐patient basis.

##### Importance of appropriate dosing of DOACS

While routine monitoring of anticoagulant effects is not required for DOACs, they must be prescribed at doses consistent with criteria established in clinical trials. Despite clear dosing reduction guidelines provided in the product information for each DOAC, inappropriate dosing—either under‐dosing or over‐dosing—remains common practice.^82^ Poli et al.^83^ investigated inappropriate DOAC dose prescription in 5943 AF; 43.1% of patients were prescribed low dose DOAC, which was associated with higher risk of major bleeding (RR 1.8; 95% CI 1.3–1.7), stroke (RR 1.3, 95% CI .7–2.4) and mortality (RR 2.8; 95% CI 1.9–4.1). Inappropriate dosing of DOACs is more prevalent among older AF patients (40% in mean age of 85.3 years old vs. 23% in mean age of 72.0 years old). This might be due to physicians' fear of DOAC‐associated bleeding in older patients.^82^, ^84^ In a meta‐analysis of 17 studies comparing off‐labelled under‐dosed DOACs versus on‐label, off‐label underdosing was associated with increased likelihood of ischaemic stroke or systemic embolism (HR 1.17; 95% CI: 1.00–1.38, *p* = .048) and ICH (1.27; 95% CI: 1.06–1.52, *p* = .010).^85^

DOAC reversal can be performed using either targeted antidotes (idarucizumab and andexanet alfa) or nonspecific haemostatic agents that support coagulation (prothrombin complex concentrate, activated prothrombin complex concentrate and recombinant factor VIIa) which work by enhancing haemostatic function.^86^

##### Bleeding risk assessment

Bleeding risk assessment involves both modifiable and nonmodifiable risk factors. The primary objective is not to withhold anticoagulation but rather to identify and address modifiable risk factors, while ensuring closer monitoring of high‐risk patients. Among the available tools, the HAS‐BLED score has emerged as the most validated and is supported by multiple systematic reviews and practical evidence.^87^, ^88^ Recent data also show the usefulness of HAS‐BLED in patients taking DOACs.^89^, ^90^, ^91^

The mAFA‐II trial provided compelling evidence for the appropriate use of the HAS‐BLED score in an integrated care approach based on the ABC pathway.^92^ In this prospective cluster randomised trial, treatment was managed based on the HAS‐BLED score as part of a mobile health intervention, resulting in significant reductions in major bleeding rates (2.1% vs. 4.3%) compared to usual care. Moreover, OAC adherence improved in the intervention group while declining in the control group, demonstrating the tool's practical value.^77^, ^78^, ^79^, ^92^

Bleeding risk assessment is not a one‐time event. Studies show that HAS‐BLED scores frequently change over time, with uncontrolled systolic blood pressure being the most common new risk factor. Follow‐up HAS‐BLED scores are more predictive of subsequent bleeding risk than baseline scores, with patients showing a 3.5‐fold higher bleeding risk in the first 3 months following a bleeding‐risk profile change. Approximately 22% of initially low‐risk patients progress to high‐risk within 1 year.^93^ Despite elevated bleeding risk scores, OAC should be maintained due to its superior net clinical benefit in high‐risk patients.

OAC resumption after ICH is always a challenging decision for physicians. In a multicentre, randomised trial among six European countries, patients with AF and spontaneous ICH were randomly assigned to receive DOACs or not receiving OACs, whereby DOACs were associated with a significant reduction in ischaemic strokes but an increased risk of ICH and other major bleeding events.^94^ A shared decision‐making approach and a tailored, individualised strategy are essential when evaluating the risks and benefits of OAC therapy in ICH survivors with AF.

The key lies in structured risk management: carefully monitoring and correcting (where possible) modifiable bleeding risk factors during each patient interaction, while implementing more intensive follow‐up protocols for those at higher risk of bleeding. For patients with significant nonmodifiable risk factors, early reassessment at 4 weeks, rather than the standard 4–6 months, enables proactive intervention and risk mitigation.^56^ This comprehensive approach focuses on risk management and patient safety while preserving the crucial benefits of anticoagulation therapy.

**FIGURE 4:**
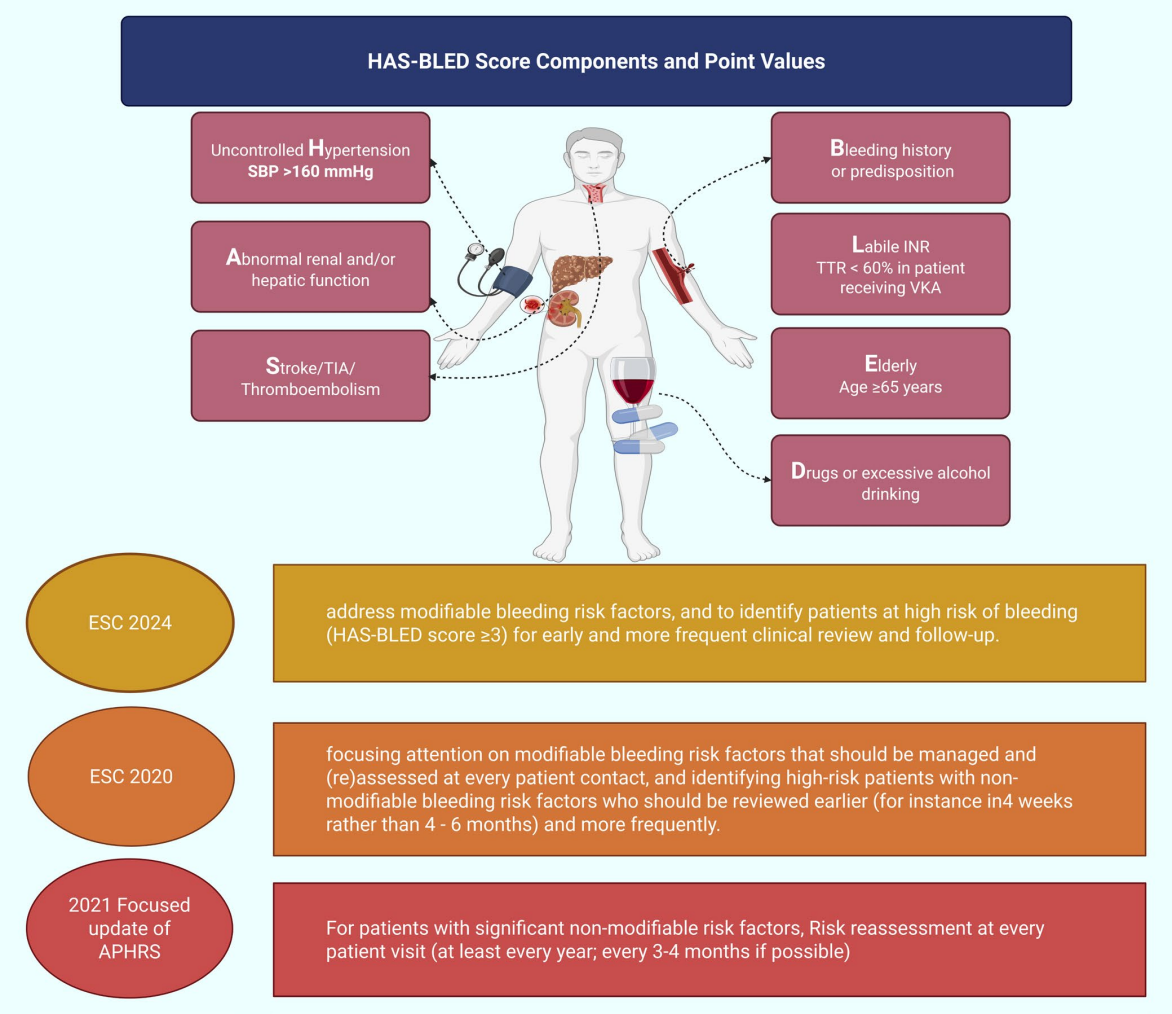
HAS‐BLED bleeding risk assessment score and recent guideline recommendations for managing atrial fibrillation (AF) patients at high risk of bleeding. The HAS‐BLED score evaluates bleeding risk based on the following factors: Hypertension (1 point), abnormal renal and/or hepatic function (1 point each, max 2), stroke history (1 point), bleeding history or predisposition (1 point), labile INRs (1 point), elderly age (≥65 years, 1 point) and drug/alcohol use (1 point each, max 2). A total score of ≥3 indicates a high risk of bleeding, warranting closer monitoring and management. The 2024^14^ and 2020 ESC guidelines,^56^ as well as the 2021 Focused update of the 2017 consensus guidelines of the Asia Pacific Heart Rhythm Society (APHRS) on stroke prevention in atrial fibrillation,^150^ recommend using the HAS‐BLED score to identify high‐risk patients for more frequent clinical review and follow‐up, in order to address modifiable bleeding factors and optimise antithrombotic management. By incorporating the HAS‐BLED assessment and guideline‐directed care, clinicians can improve outcomes for AF patients at heightened risk of bleeding complications.

Table 4 and Figure 4 present recent guideline recommendations for enhanced follow‐up strategies in managing AF patients according to HAS‐BLED score.

**TABLE 4:** Recent guidelines on enhanced follow‐up strategies for managing atrial fibrillation patients with high bleeding risk.

Guideline	Recommendations
2024 ESC Guidelines^14^	Use HAS‐BLED Score to address modifiable bleeding risk factors. (Class IIa, level B) Identify high‐risk patients (HAS‐BLED score ≥3) for early and frequent follow‐up. (Class IIa, level B)
2020 ESC Guidelines^56^	Focus on managing modifiable risk factors at every patient contact. (Class IIa) Identify high‐risk patients with nonmodifiable risk factors for earlier (e.g. 4 weeks) and more frequent reviews. (Class IIa)
2021 Focused update of the 2017 consensus guidelines of the APHRS^150^	Perform risk reassessment at every patient visit: At least annually Preferably every 3–4 months

Abbreviations: APHRS, Asia Pacific Heart Rhythm Society; EACTS, European Association of Cardio‐Thoracic Surgery; ESC, European Society of Cardiology; HAS‐BLED, hypertension, abnormal renal and liver function, stroke, bleeding history or predisposition, labile INR, elderly, drugs or alcohol use; OAC, oral anticoagulants.

##### Contraindications for DOACS

DOACs are contraindicated in pregnancy, mechanical heart valves, moderate‐to‐severe mitral stenosis and end‐stage renal disease. In these conditions, VKAs remain the treatment of choice for anticoagulation.^95^

*Pregnancy*. DOACs are contraindicated during pregnancy, and women of childbearing age must use effective contraception before initiating DOAC therapy. In this population, low‐molecular‐weight heparin is the preferred anticoagulant as it does not cross the placenta.^96^

*Patients with end‐stage CKD or on dialysis*. DOACs are considered effective and safe alternatives to VKAs in patients with mild‐to‐moderate chronic kidney disease (CKD) (creatinine clearance (CrCl) >30 mL/min), provided appropriate dose adjustments are made.^97^ In Europe, lower doses of rivaroxaban, apixaban and edoxaban have been approved for patients with severe CKD (CrCl 15–29 mL/min), although these approvals are supported by limited data from major RCTs comparing DOACs to VKAs^98^ and should be used with caution. In contrast, dabigatran, which is heavily dependent on renal clearance, is contraindicated in patients with a CrCl <30 mL/min/1.73 m^2^.

In Europe, the use of rivaroxaban, apixaban and edoxaban is not approved for patients with a CrCl below 15 mL/min or those on dialysis.^76^ In contrast, apixaban and rivaroxaban are approved for patients on dialysis in the USA. The American AF guidelines provide a Class IIb recommendation suggesting that warfarin (target INR 2.0–3.0) or apixaban may be considered for OAC in patients with AF and CrCl <15 mL/min or those undergoing dialysis.^44^

Evidence from small studies in patients on haemodialysis has been varied. Two trials showed no significant differences in efficacy or safety between apixaban (2.5 mg twice daily) and VKAs,^99^, ^100^ whereas another study found that rivaroxaban (10 mg) led to significantly lower rates of cardiovascular events (HR .41, 95% CI .25–.68; *p* = .0006) and major bleeding (HR .39, 95% CI .17–.90; *p* = .03) compared to VKAs.^101^ Careful assessment of individual patient factors and regular follow‐up are essential when prescribing anticoagulants to those with compromised renal function.

A recent meta‐analysis of the four RCTs (486 dialysis‐dependent AF patients, with a median follow‐up of 26 weeks to 1.88 years) demonstrated that when compared to VKAs, Factor Xa inhibitors were associated with a reduced risk of major bleeding (RR = .64, 95% CI = .42–.99, *p* = .04), but no significant difference in stroke and systemic embolism (RR = .46, 95% CI = .20–1.02, *p* = .06).^102^ Factor Xa inhibitors also showed a significantly lower risk of intracranial bleeding (RR = .40, 95% CI = .17–.96, *p* = .04), but no significant differences were evident for other outcomes, including gastrointestinal bleeding, hemorrhagic stroke, ischaemic stroke, acute coronary syndrome and mortality.^102^ Hence, the oral Factor Xa inhibitors may offer a safer alternative to VKAs for AF patients on dialysis, with a lower risk of bleeding and similar risks of stroke and mortality.

Several ongoing large‐scale RCTs aim to evaluate OAC use in patients with AF undergoing dialysis. The AVKDIAL (NCT02886962) and DANWARD (NCT03862859) trials are comparing VKA therapy to no OAC. The SACK trial (NCT05679024) examines apixaban 2.5 mg twice daily versus no OAC. Meanwhile, the SAFE‐D trial (NCT03987711) evaluates apixaban (5 mg or 2.5 mg twice daily for select patients), warfarin and no OAC. These studies aim to clarify the net clinical benefit of OACs and guide optimal anticoagulant selection in this high‐risk population.

*Patients with moderate‐to‐severe mitral stenosis*. Observational studies of DOAC use in patients with moderate‐to‐severe mitral stenosis have demonstrated promising outcomes, with the DOAC group showing a greater reduction in ischaemic stroke or systemic embolism compared to the warfarin group.^103^, ^104^ Building on these findings, Sadeghipour et al. conducted a randomised pilot trial on patients with moderate‐to‐severe mitral stenosis (*n* = 40), which further supported the efficacy and safety of rivaroxaban use.^105^

However, the recent INVICTUS trial (Investigation of Rheumatic AF Treatment Using Vitamin K Antagonists, Rivaroxaban or Aspirin Studies), which enrolled 4531 patients with AF and rheumatic heart disease (predominantly mitral stenosis in 85% of participants), found that VKA therapy resulted in a lower rate of composite cardiovascular events or death compared to rivaroxaban (446 vs. 560 events; difference in restricted mean survival time: −76 days; 95% CI, −121 to −31; *p* < .001), with a higher incidence of death in the rivaroxaban group (restricted mean survival time: 1608 vs. 1680 days; difference, −72 days; 95% CI, −117 to −28).^106^

*Challenges in management of atrial fibrillation in cancer patients*. Recent studies have indicated that there is a considerable risk of AF with a prevalence of 2%–28% in all cancer types specially among older patients with pre‐existing cardiovascular disorders.^107^, ^108^ AF patients with cancer have a two‐fold higher risk of thromboembolic events and stroke as well as an increased risk of mortality and major bleeding.^108^, ^109^, ^110^ Considering the increased risk of thromboembolic events and major bleeding as a complication of cancer and OACs, there is a need for specific risk assessment tools rather than conventional risk scores such as CHA_2_DS_2_‐VASc and HAS‐BLED scores which may not completely evaluate the dynamic risk of stroke and bleeding in this specific population. Multi‐disciplinary decision making with cardio‐oncologists, cardiologists and haematologists is needed for optimum management.

*New and emerging options for anticoagulation*. Factor XI inhibitors might be a new option in future for stroke prevention in patients with AF. PACIFIC‐AF, a phase 2, multicentre, randomised, double‐blind trial recruiting 765 AF patients among 14 countries, demonstrated that 20 mg and 50 mg asundexian once daily were associated with a significant reduction of bleeding events compared with standard dosing of apixaban (incidence ratio .33, 90% CI .90–.97).^111^ The ongoing LIBREXIA‐AF trial is evaluating if milvexian is noninferior to apixaban in its effectiveness for decreasing the risk of the composite stroke and peripheral embolism in AF patients.^112^ If we observe the promising results from the aforementioned trials, the landscape of AF stroke prevention would change significantly.

*Left atrial appendage occlusion or closure*. In AF patients who are unable or unwilling to use OACs, left atrial appendage occlusion (LAAO) presents a promising alternative for stroke prevention.^43^ There are several reasons why OAC may not be appropriate, including the risk of bleeding, poor adherence or resistance to treatment. Additionally, patients with contraindications to anticoagulation therapy, such as recent ICH, intractable gastrointestinal bleeding or advanced renal failure, may benefit from nonpharmacological interventions like LAAO. Furthermore, stroke events may persist despite appropriate anticoagulation, and in these cases, LAAO has been proposed as a viable solution.^113^

The left atrial appendage (LAA) is the primary site for thrombus formation in AF, responsible for approximately 90% of thromboembolic events.^114^ Observational studies have demonstrated the benefits of LAA ligation during cardiac surgery, but since most AF patients do not require surgery, percutaneous LAAO was introduced as an effective alternative.^115^, ^116^ An RCT comparing concomitant LAAO with usual OAC therapy versus OAC therapy alone demonstrated a significant reduction in the composite outcome of stroke or systemic embolism (HR .67; 95% CI .53–.85); incidence of heart failure, perioperative bleeding and death were comparable among groups.^117^ Nevertheless, ongoing studies comparing LAAO to DOACs suggest that LAAO might result in superior clinical outcomes due to its' potential for reducing bleeding complications.

In the landmark OPTION trial (Comparison of Anticoagulation with Left Atrial Appendage Closure after AF Ablation) of 1600 patients with AF and elevated stroke risk, LAA closure was compared to OAC. LAA closure significantly reduced nonprocedure‐related bleeding events (8.5% vs. 18.1%, *p* < .001) while maintaining similar efficacy in preventing stroke, systemic embolism and death (5.3% vs. 5.8%, *p* < .001 for noninferiority). Major bleeding rates, including procedure‐related events, were comparable between groups (3.9% vs. 5.0%, *p* < .001 for noninferiority), suggesting LAA closure as a viable alternative to long‐term anticoagulation in eligible patients.^118^ In the PRAGUE‐17 trial, a multicentre, randomised, noninferiority trial comparing LAAO with DOACs, the risk of stroke or TIA (HR 1.00, 95% CI: .40–2.51), clinically significant bleeding (HR .81, 95% CI: .44–1.52) and cardiovascular mortality (HR .75, 95% CI .34–1.62) were comparable between groups after a median 19.9 months of follow‐up.^119^

In addition to trials, several registries, including the EWOLUTION (registry on WATCHMAN outcomes in real‐life utilisation) study^120^ and the ASAP (ASA Plavix Feasibility Study With Watchman Left Atrial Appendage Closure Technology) study,^121^ have evaluated the efficacy of LAAO in patients with contraindications to OAC therapy. These observational cohorts demonstrate a significant reduction in stroke rates and bleeding compared to expected outcomes based on the CHA_2_DS_2_‐VASc and HAS‐BLED scores, although such comparisons have major limitations given the different populations and clinical settings. For example, the EWOLUTION registry observed an 83% relative risk reduction in stroke and a 46% relative risk reduction in major nonprocedural bleeding rates at 2‐year follow‐up in patients with contraindications to OAC.

A network meta‐analysis of observational and trial data indicated that while LAAO may be slightly less effective than DOACs in preventing ischaemic stroke, it offers significant protection against major and life‐threatening bleeding.^122^, ^123^ This benefit persists throughout the treatment duration and may become increasingly relevant over time when compared to the ongoing risks associated with lifelong DOAC therapy.

Importantly, shared decision‐making between clinicians and patients plays a vital role in determining the best therapeutic approach. In addition to considering the clinical efficacy of LAAO, it is essential to account for the patient's quality of life, lifestyle preferences and overall satisfaction with the chosen treatment. Some patients may prefer LAAO to avoid long‐term anticoagulation therapy, especially in those who participate in high‐risk activities or experience significant anxiety about bleeding events.

Ongoing trials evaluating the safety and efficacy of LAAO are summarised in Table 5. These trials aim to further clarify the role of LAAO in stroke prevention, especially in patients with ICH, advanced CKD or those who have failed anticoagulation therapy.

**TABLE 5:** Randomised controlled trials evaluating safety and efficacy of LAAO in AF patients.

Title	ClinicalTrials.gov Identifier	Primary outcome
COMPARE‐LAAO (Comparing Effectiveness and Safety of Left Atrial Appendage Occlusion for Non‐valvular Atrial Fibrillation Patients at High Stroke Risk Unable to Use Oral Anticoagulation Therapy)	NCT04676880	(1) Time to first occurrence of stroke (ischaemic, hemorrhagic or of unknown origin) and (2) time to first occurrence of a composite outcome consisting of stroke, transient ischaemic attack and systemic embolism.
ASAP TOO (Assessment of the WATCHMAN™ Device in Patients Unsuitable for Oral Anticoagulation) trial	NCT02928497	Comparison of time to first event of ischaemic stroke and systemic embolism in 5 years.
STROKECLOSE (Prevention of stroke by left atrial appendage closure in AF stroke patients with intracerebral Haemorrhage) trial	NCT02830152	Composite endpoint of stroke (ischaemic and hemorrhagic), systemic embolism, life‐threatening or major bleeding and all‐cause mortality, assessed over at least 2 years.

There are a variety of post‐LAAO antithrombotic regimens to avoid device‐related thrombosis and associated thromboembolic events. Some studies suggest 45 days of VKA plus aspirin after implantation, followed by 6 months of dual anti‐platelet therapy.^124^, ^125^ In a propensity‐score matched cohort of patients receiving either OAC (*n* = 1018; 95% on warfarin, 5% on nonwarfarin OAC) or antiplatelet therapy (*n* = 509; 91% on dual antiplatelet therapy, 9% on single antiplatelet therapy), 6‐month freedom from nonprocedural major bleeding and thromboembolism beyond 7 days were comparable between the two groups. However, device‐related thrombosis occurred more frequently in the antiplatelet therapy group compared to OAC (OAC: 1.4% vs. antiplatelet therapy: 3.1%; *p* = .018).^126^ In a meta‐analysis (16 studies, 3255 patients), the incidence of stroke (RR 1.33, 95% CI .64–2.77), device‐related thrombosis (RR 1.52, 95% CI .90–2.58), and the combined outcome of stroke and device‐related thrombosis (RR 1.26, 95% CI .67–2.37) showed no significant difference between the single antiplatelet therapy and dual antiplatelet therapy groups.^127^

There are two ongoing randomised controlled trials (NCT03445949 and NCT03568890) evaluating the most appropriate post‐LAAO antithrombotic regimens, comparing 30 days versus 6 months of dual antiplatelet therapy and discontinuation of all antithrombotic therapy versus continuation of long‐term single antiplatelet therapy in the SAFE‐LAAC trial (NCT03445949) and anticoagulation versus antiplatelet therapy in the ANDES trial (NCT03568890), respectively.

In summary, while OAC remains the first‐line therapy for stroke prevention in AF, LAAO offers a promising alternative for patients with contraindications or resistance to OAC therapy. LAAO may offer long‐term benefits, particularly in reducing bleeding risks and improving clinical outcomes for patients who cannot tolerate or adhere to pharmacological treatments. Further research and clinical trials are essential to refine its role in stroke prevention.

### 4.2 Improving stroke outcomes through rate and rhythm management

Better patient‐centred, symptom‐directed tailored rate or rhythm management strategies are primarily aimed to improve quality of life in AF patients, but it also indirectly contributes to stroke prevention by reducing the risk factors that lead to thromboembolism. Recent studies have provided quantitative evidence supporting the impact of early rhythm control on improving outcomes, including reductions in stroke rates.^128^, ^129^

In managing AF, two main approaches are commonly used. Rate control focuses on slowing the ventricular rate to a level that is physiologically appropriate. This strategy is favoured for its simplicity and the avoidance of potential side effects associated with anti‐arrhythmic drugs, electrical cardioversion or invasive procedures like left atrial ablation for recurrent AF. On the other hand, rhythm control aims to restore and maintain sinus rhythm over the long term. This is typically achieved through the use of anti‐arrhythmic drugs, particularly ion channel blockers. In some cases, autonomic manipulation, such as using beta blockers, can also play a role in managing the condition.

Effective rate or rhythm control plays a critical role in maintaining atrial function and minimising blood stasis in the left atrium and LAA, key sites for thrombus formation in AF. Rhythm control interventions, such as antiarrhythmic drug therapy or catheter ablation, have demonstrated a reduction in AF burden. This translates into decreased risk of thromboembolism. A post hoc analysis of the Atrial Fibrillation Follow‐up Investigation of Rhythm Management (AFFIRM) trial revealed that AF patients managed using the ABC pathway experienced significantly better outcomes, including reduced risk of all‐cause mortality (HR .35; 95% CI .17–.75), composite outcomes of stroke, major bleeding, cardiovascular death and first hospitalisation (HR .35; 95% CI .18–.68), and first hospitalisation (HR .65; 95% CI .53–.80). Notably, patients who adhered to the ‘B’ and ‘C’ criteria of the ABC pathway also demonstrated significantly improved outcomes compared to those who did not adhere to the ABC pathway strategy.^47^

Recent studies highlight the advantages of rhythm control in AF management in reducing stroke or TIA risk (HR, .80, 95% CI .74, .87).^130^ The CABANA trial demonstrated a significant reduction in AF recurrence(HR .52, 95% CI .45–.60) and cardiovascular hospitalisation (HR .83, 95% CI .74–.93) associated with catheter ablation.^129^ The EAST‐AFNET trial demonstrated that early rhythm control reduces the composite endpoint of cardiovascular death, stroke or hospitalisation (HR .79, 95% CI, .66–.94), and RAFAS reported lower IS recurrence at 12 months (HR .25, 95% CI .06–1.00).^128^, ^131^ The AFFIRM sub‐study noted no differences between rate and rhythm control in outcomes but highlighted benefits in those with new‐onset AF.^132^ The EAST‐AFNET 4 trial study population was patients who were diagnosed with AF in the past 12 months and had cardiovascular comorbidities, suggesting that early rhythm control might be more beneficial in newly diagnosed patients with comorbidities.^128^ A healthy lifestyle is associated with more efficiency of early rhythm control in young AF patients.^133^

However, further randomised studies are needed to evaluate this approach in more diverse populations of AF patients to enhance the generalizability and applicability of early rhythm control intervention. Taken together, these findings strongly reinforce the role of rhythm control as an effective strategy for reducing stroke risk (Table 6).

**TABLE 6:** Summary of studies on rhythm versus rate control in atrial fibrillation.

First author, publication year	Sample size	Study design	Main finding
Tsadok, 2012^130^	16,325 patients on rhythm control therapy (with or without rate control) and 41,193 on rate control therapy.	Prospective cohort	Rhythm control therapy (anti‐arrhythmic drugs) was associated with a lower rate of stroke/TIA compared to rate control therapy (1.74 vs. 2.49 per 100 person‐years, *p* < .001).
Packer, 2019^129^	2204 patients with atrial fibrillation, catheter ablation (*n* = 1108), compared with medical therapy (*n* = 1096).	Randomised controlled trial	Composite of death, disabling stroke, serious bleeding or cardiac arrest: Ablation group: 8.0% (89 patients). Drug therapy group: 9.2% (101 patients). HR: .86 (95% CI, .65–1.15); *p* = .30 (not statistically significant). All‐cause mortality: Ablation group: 5.2%. Drug therapy group: 6.1%. HR: .85 (95% CI, .60–1.21); *p* = .38 (not statistically significant). Death or cardiovascular hospitalisation: Ablation group: 51.7%. Drug therapy group: 58.1%. HR: .83 (95% CI, .74–.93); *p* = .001 (statistically significant). Atrial fibrillation recurrence: Ablation group: 49.9%. Drug therapy group: 69.5% HR: .52 (95% CI, .45–.60); *p* < .001 (statistically significant).
Kirchhof, 2020^128^	2789 patients with early atrial fibrillation	Randomised controlled trial	Composite of cardiovascular death, stroke or hospitalisation for worsening heart failure or acute coronary syndrome: Early rhythm control: 249 events (3.9 per 100 person‐years). Usual care: 316 events (5.0 per 100 person‐years). HR: .79 (95% CI, .66–.94); *p* = .005 (statistically significant). Hospitalisation: Mean nights in hospital per year: ○ Early rhythm control: 5.8 ± 21.9 nights. ○ Usual care: 5.1 ± 15.5 nights. *p* = .23 (not statistically significant). Composite of death, stroke or serious adverse events related to rhythm‐control therapy: No significant difference between groups. Serious adverse events related to rhythm‐control therapy: ○ Early rhythm control: 4.9%. ○ Usual care: 1.4%. Symptoms and left ventricular function: No significant differences between groups at 2 years.
Park, 2022^131^	300 patients with AF and an acute IS randomised 2:1 to early rhythm control (*n* = 194) or usual care (*n* = 106).	Randomised controlled trial	IS recurrences (within 3 months of the index stroke): Early rhythm control group: 2 events (1.1%). Usual care group: 4 events (4.2%). HR: .257 (log‐rank *p* = .091) (not statistically significant). IS recurrences (at 12 months): Early rhythm control group: 3 events (1.7%). Usual care group: 6 events (6.3%). HR: .251 (log‐rank *p* = .034) (statistically significant). Overall mortality: Early rhythm control group: 25 events (14.0%). Usual care group: 16 events (16.8%). HR: .808 (log‐rank *p* = .504) (not statistically significant). Any cause of hospitalisations: Early rhythm control group: 25 events (14.0%). Usual care group: 16 events (16.8%). HR: .808 (log‐rank *p* = .504) (not statistically significant). Arrhythmia‐related adverse events: Early rhythm control group: 5 events (2.8%). Usual care group: 1 event (1.1%). HR: 2.565 (log‐rank *p* = .372) (not statistically significant). Sustained atrial fibrillation (at 12 months): Early rhythm control group: 60 patients (34.1%). Usual care group: 59 patients (62.8%). *p* < .001 (statistically significant).
Yang, 2021^132^	Post hoc analysis	4060 AFFIRM subjects	Risk of all‐cause mortality: Individuals with ‘new’ AF (diagnosed within 6 months) had a decreased risk of all‐cause mortality compared to those with prior AF diagnoses (*p* = .001). Demographics and comorbidities: Participants with prior AF were similar in age and demographics but had higher rates of medical comorbidities, including: ○ Myocardial infarction: *p* = .006 ○ Diabetes mellitus: *p* = .002 ○ Smoking: *p* = .003 ○ Hepatic or renal comorbidities: *p* = .008 Outcomes (rate vs. rhythm control): No significant differences in: ○ Mortality ○ Cardiovascular hospitalisations ○ Stroke These results were consistent across both AF subgroups (new vs. prior AF).

Abbreviations: AF, atrial fibrillation; AFFIRM, Atrial Fibrillation Follow‐up Investigation of Rhythm Management; CABANA, catheter ablation versus antiarrhythmic drug therapy for atrial fibrillation; CI, confidence interval; EAST‐AFNET, early treatment of atrial fibrillation for stroke prevention trial; HR, hazard ratio; RAFAS, risks and benefits of early rhythm control in patients with acute strokes and atrial fibrillation study; TIA, transient ischaemic attack.

### 4.3 Cardiovascular risk factor management and stroke prevention

AF is influenced by both the condition itself and various cardiovascular comorbidities, risk factors and unhealthy lifestyles, all of which contribute to adverse atrial remodelling. Early rhythm control interventions aim to prevent AF‐associated remodelling and reduce the risk of cardiovascular events such as stroke. However, this approach is most effective when paired with comprehensive management of comorbidities and lifestyle optimisation, as emphasised in the ‘C’ component of the ABC pathway.

Evidence from the RACE 3 trial supports this approach, demonstrating that addressing comorbidities and risk factors in patients with persistent AF and mild‐to‐moderate heart failure reduced AF burden and improved sinus rhythm maintenance in the intervention group compared to the conventional group (odds ratio 1.76, 95% CI 1.02–3.05).^134^ However, long‐term success requires sustained efforts, as benefits diminished at 5‐year follow‐up. Similarly, lifestyle modifications, such as weight loss and managing clustered behaviours like smoking, alcohol use and poor exercise, have shown significant reductions in AF burden and severity.^135^, ^136^ Adherence to such interventions is critical, as clustering of unhealthy habits increases the risk of major cardiovascular events, including ischaemic stroke.

Multimorbidity is prevalent in AF patients, with 80%–90% experiencing two or more chronic conditions, a much higher rate than in the general population.^137^, ^138^ This underscores the importance of integrated, multidisciplinary care pathways like the ABC approach, which target comorbidities, risk factors and lifestyle modifications simultaneously. Randomised controlled trials addressing multiple factors, rather than isolated conditions, have shown improved outcomes, including reduced AF symptoms and enhanced rhythm control success. Despite these advances, long‐term adherence to structured, multidisciplinary interventions remains challenging, particularly in multimorbid patients. Ongoing studies, such as the EU Horizon AFFIRMO and EHRA‐PATHS projects, aim to refine and optimise management strategies, leveraging tools like AI to tailor treatments for complex patients. Holistic, evidence‐based care remains essential for improving outcomes in AF, emphasising a shift from single‐condition management to integrated, patient‐centred approaches.^51^, ^118^, ^139^

## 5 CONCLUSION

OACs, especially DOACs, remain the primary treatment for stroke prevention in AF, although LAAO is emerging as a viable option for patients who cannot use long‐term anticoagulation. Clinical evidence also supports the role of early rhythm control in reducing stroke risk in patients with recent‐onset AF.

The ABC pathway approach offers a clear framework for AF management. First, assess whether stroke prevention is needed and prescribe OAC as appropriate. Then, make decisions focused on the patient's symptoms about using either rate or rhythm control treatments. Finally, tackle cardiovascular risk factors, manage other health conditions and encourage lifestyle changes.^35^ Adherence to the ABC pathway, which integrates comprehensive care strategies, has been shown through both trial and cohort studies to improve clinical outcomes, including a reduction in stroke risk and other complications. Additionally, digital health technologies are becoming increasingly essential in the diagnosis and management of AF, offering the potential to improve outcomes in stroke prevention with timely diagnosis and screening. Future directions in AF stroke prevention should focus on wearable devices and AI‐driven ECG monitoring for earlier AF detection, machine learning‐based stroke risk prediction models for improving timely intervention and personalised anticoagulation for minimising the bleeding risk ensuring the effective stroke prevention.

## AUTHOR CONTRIBUTIONS

Amir Askarinejad was involved in visualisation, writing—original draft and writing—review and editing. Deirdre A Lane, Parham Sadeghipour and Majid Haghjoo were involved in writing—review and editing. Gregory Y. H. Lip was involved in conceptualisation, project administration and supervision.

## CONFLICT OF INTEREST STATEMENT

Deirdre A. Lane received investigator‐initiated educational grants from Bristol‐Myers Squibb and Pfizer, outside the submitted work. She is a co‐applicant on the AFFIRMO project on multimorbidity in AF (Grant Agreement No. 899871), ARISTOTELES project on artificial intelligence for management of chronic long‐term conditions (Grant Agreement No. 101080189), and the TARGET project on digital twins for personalised management of atrial fibrillation and stroke (Grant Agreement No. 101136244), all of which are funded by the European Union Horizon Europe Research and Innovation programme.

## DECLARATION OF GENERATIVE AI AND AI‐ASSISTED TECHNOLOGIES IN THE WRITING PROCESS

During the preparation of this work, the authors used Claude 2.1 and ChatGPT 4o for improving English language fluency and ensuring native quality writing. Claude was consulted regarding grammar, word choice, sentence structure and overall clarity of expression. After using this service, the authors reviewed and edited the content as needed and took full responsibility for the content of the publication.

## Data Availability

Data sharing not applicable—no new data generated.
